# Quantification of *Salmonella* Survival and Infection in an *In vitro* Model of the Human Intestinal Tract as Proxy for Foodborne Pathogens

**DOI:** 10.3389/fmicb.2017.01139

**Published:** 2017-06-30

**Authors:** Lucas M. Wijnands, Peter F. M. Teunis, Angelina F. A. Kuijpers, Ellen H. M. Delfgou-Van Asch, Annemarie Pielaat

**Affiliations:** ^1^National Institute of Public Health and the EnvironmentBilthoven, Netherlands; ^2^Rollins School of Public Health, Emory UniversityAtlanta, GA, United States

**Keywords:** model-system, GI-tract, infection, foodborne pathogens, Bayesian, quantification

## Abstract

Different techniques are available for assessing differences in virulence of bacterial foodborne pathogens. The use of animal models or human volunteers is not expedient for various reasons; the use of epidemiological data is often hampered by lack of crucial data. In this paper, we describe a static, sequential gastrointestinal tract (GIT) model system in which foodborne pathogens are exposed to simulated gastric and intestinal contents of the human digestive tract, including the interaction of pathogens with the intestinal epithelium. The system can be employed with any foodborne bacterial pathogens. Five strains of *Salmonella* Heidelberg and one strain of *Salmonella* Typhimurium were used to assess the robustness of the system. Four *S*. Heidelberg strains originated from an outbreak, the fifth *S*. Heidelberg strain and the *S*. Typhimurium strain originated from routine meat inspections. Data from plate counts, collected for determining the numbers of surviving bacteria in each stage, were used to quantify both the experimental uncertainty and biological variability of pathogen survival throughout the system. For this, a hierarchical Bayesian framework using Markov chain Monte Carlo (MCMC) was employed. The model system is able to distinguish serovars/strains for *in vitro* infectivity when accounting for within strain biological variability and experimental uncertainty.

## Introduction

Dose response (DR) assessment is that part of the Quantitative Microbial Risk Assessment (QMRA) framework in which exposure to pathogenic microorganisms is translated into human health risk. DR assessment based on human clinical experiment data is most common (Teunis et al., 1996). For pathogens that cannot be tested in humans, because they cause severe or long term health effects, animal data have been used (Havelaar et al., 2001; Stecher et al., 2005). Due to the specificity of the host-pathogen relation, translation of animal results to the human host is difficult (Berk, 2008) and use of an animal proxy may be problematic (Haas et al., 2000). For a few pathogens, data from outbreaks can be found, that allow analysis as a “natural experiment,” to infer a DR relation (Teunis et al., 2008, 2010). Although in an outbreak the number of cases is usually known, the number of exposed subjects and the number of pathogens in the food (i.e., the dose) are rarely reported.

Infection and illness endpoints are influenced by host factors and pathogen factors. Clinical challenge data are biased by the selection of healthy (young) adults for such studies, and selection of less virulent pathogens. It is generally understood that foodborne disease preferably occurs in vulnerable groups, described with the term YOPIs: the young, the old, the pregnant, and the immunocompromised. Therefore, human challenge studies may not reflect the DR as would occur in the general population (Kothary and Babu, 2001). Moreover, different isolates of the same pathogen species have been shown to have strongly different infectivities and/or pathogenicities (Haas et al., 2000; Teunis et al., 2002, 2004, 2010; Strachan et al., 2005). The effects of pathogen and host factors may be quantified in a DR model.

This variability in host and pathogen factors explains why it is difficult to study DR on a high aggregation level. Therefore, in this study we used a “simple” test system to gain DR knowledge.

**Figure 1:**
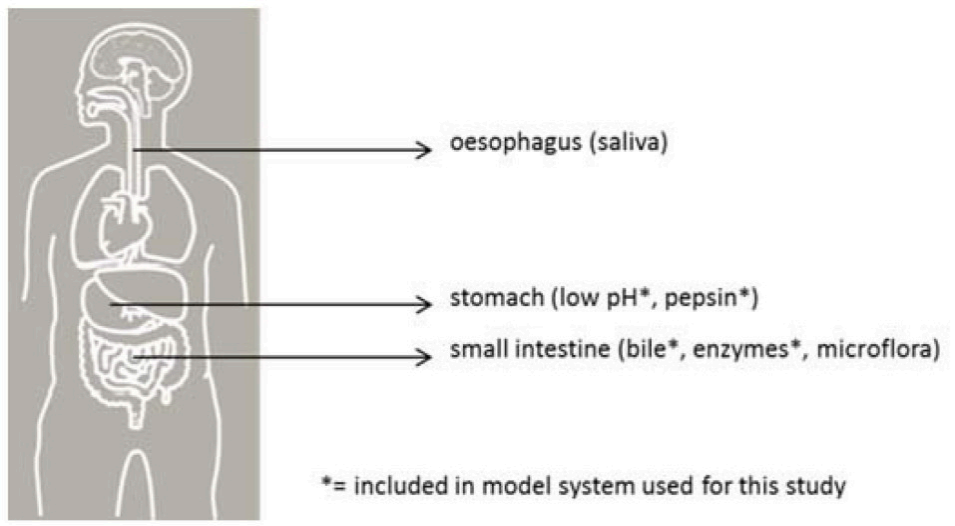
Barriers for pathogenic bacteria in the human gastrointestinal tract. The sites are indicated with their respective barrier indicators.

Before reaching a site in the host suitable for colonization, inoculated pathogens must pass multiple barriers, each with a varying probability of survival (see Figure 1). *In vitro* models simulating human gastrointestinal passage can be used to study some of these barriers as a partial model for infection DR in humans. The survival of various pathogens can be quantified and compared in a standardized intestinal tract model and, with that, provide insight into the relative risk of these pathogens for human health.

This study has two objectives. Firstly, we describe in detail a gastrointestinal tract (GIT) model system consisting of several stages. Bacteria from an overnight stationary culture are exposed sequentially, i.e., transfer from one stage to the next without intermediate culturing, to simulated gastric fluid and simulated intestinal fluid (SIF). Next, the interaction of the surviving bacteria with a confluent culture of cells, mimicking the human small intestinal epithelium, is studied. Both attachment and invasion are assessed in this stage. The survival of bacteria through each stage of the GIT system is monitored by plate counting samples, and the fraction of inoculated bacteria invading the intestinal cells is interpreted as a quantitative measure for human intestinal infection. The system is not meant to provide absolute DR data, but the resulting fractions of various strains/serovars can be compared to determine relative risks.

This simulation system for gastrointestinal passage involves many different parameters. An important requisite for the implementation of such a system for research purposes is experimental reproducibility, not just on the same day, but also between days.

Therefore, the second objective of this study is to quantify both the experimental uncertainty and biological variability of the test system, such that (1) the system can be improved to reduce experimental uncertainty and (2) both uncertainty and variability can be quantified when describing the characteristics of different gastro-intestinal bacterial pathogens in future applications of the GIT system. Insight in the biological variation in relative risk of passage and infection by different foodborne pathogens provides insight into the influence of pathogen factors on the dose response relation for human infection. This information, in turn, is important for prioritizing intervention measures, for instance. To meet this second objective, two serovars of *Salmonella enterica* var. *enterica*, namely Heidelberg and Typhimurium, were run through the system several times, at different days and on 1 day.

## Materials and methods

### Bacterial strains and culture conditions

For the present study, we used five strains of *Salmonella* Heidelberg (SH) and one strain of *Salmonella* Typhimurium (STM). Of the SH strains, four originated from an outbreak in The Netherlands (Van Rijckevorsel et al., 2015) and one from poultry meat (strain 980). One of the outbreak strains (1043) was isolated from a sample of the implicated food (a pasta meal), the other three strains (1007, 1011 and 1028) were isolated from patient feces. All strains were provided by the Center for Infectious Diseases, Epidemiology and Surveillance of the National Institute for Public Health and the Environment (IDS/RIVM). The strains were stored at −70°C on porous beads (Microbanks, Pro-Lab, The Netherlands). For investigation in the model system, beads were cultured overnight at 37°C on Columbia agar with sheep blood (Oxoid, United Kingdom) and from there on one colony was cultured in Brain Heart Infusion broth (BHI, bioTRADING Benelux B.V., The Netherlands).

Surviving bacteria in each stage in the model system were enumerated after serial 10-fold dilution of samples in peptone-physiological-salt solution (PPS) and subsequent plating in duplicate on Trypton Soy Agar (TSA, bioTRADING Benelux B.V., The Netherlands). Plates were incubated overnight at 37°C before reading.

### Simulated gastrointestinal fluids

The composition of simulated gastrointestinal fluid (SGF) and simulated intestinal fluid (SIF) was based on previously described methods (Rotard et al., 1995; Oomen et al., 2003). The preparation of the SGF and SIF was based on the description by Oliveira et al. (2011). In more detail, SGF consisted of sodium chloride (175.0 g/L), sodium dihydrogen phosphate (88.8 g/L), potassium chloride (89.6 g/L), calcium chloride (22.2 g/L), ammonium chloride (30.6 g/L), glucose (65.0 g/L), glucuronic acid (2.0 g/L), urea (25.0 g/L), glucosamine (33.0 g/L), bovine serum albumin fraction V (1.0 g/L), mucin (Type II from porcine stomach) (3.0 g/L) and pepsin (1.3 g/L). The pH was set at the desired value with hydrochloric acid (1.0 mol/L). Mucin and pepsin, both radiation sterilized, were added after filter-sterilization, and the final SGF was mixed overnight at room temperature. In regular experiments, the pH of SGF was set at 2.5 with hydrochloric acid (1.0 mol/L).

SIF-complete consisted of two solutions: SIF-basic and bile-solution. SIF-basic consisted of sodium chloride (175.3 g/L), sodium bicarbonate (84.7 g/L), potassium dihydrogen phosphate (8.0 g/L), potassium chloride (89.6 g/L), magnesium chloride (5.0 g/L), urea (25.0 g/L), calcium chloride dehydrate (29.8 g/L), bovine serum albumin fraction V (1.0 g/L), lipase (0.5 g/L) and pancreatin (3.0 g/L). The pH was set at 7.8 ± 0.2 with hydrochloric acid (1.0 mol/L) and/or sodiumhydroxide (1.0 mol/L). Lipase and pancreatin, both radiation sterilized, were added after filter-sterilization of the rest of the solution. Basic SIF was mixed overnight at room temperature.

Bile-solution consist of sodium chloride (175.3 g/L), sodium bicarbonate (84.7 g/L), potassium chloride (89.6 g/L), urea (25.0 g/L), calcium chloride dehydrate (29.8 g/L), bovine serum albumin fraction V (1.8 g/L) and bile (6.0 g/L). The pH was set at 8.0 ± 0.2 with hydrochloric acid (1.0 mol/L) and/or sodiumhydroxide (1.0 mol/L). Bile, radiation sterilized, was added after filter-sterilization, and the bile solution was mixed overnight at room temperature.

SIF-complete was prepared by mixing 3 parts SIF-basic with 1 part bile solution. All reagents were from Merck (Germany) except, ammonium chloride, calcium chloride dihydrate, glucuronic acid, lipase and bile (Sigma, St. Louis, USA).

### Caco-2 cell culture

Caco-2 cells were cultured and differentiated, essentially as described before (Oliveira et al., 2011). In more detail, Caco-2 cells, obtained from the American Type Culture Collection (ATCC, HTB-37, USA), were routinely maintained in Dulbecco's Modified Eagle's Medium (DMEM, Gibco, Scotland) supplemented with 10% heat-inactivated fetal bovine serum (FBS, Gibco, Scotland), 1% non-essential amino acids (Gibco), 1% glutamine 100× (Gibco) and 0.1% gentamicin (50.0 mg/mL, Gibco) in 75 cm^2^ flasks (Corning Inc., USA). The cells were grown to confluence (ca. 1.0 × 10^6^ cells mL^−1^, 7 days) at 37°C in a humidified atmosphere of 95% air and 5% CO_2_. Differentiation of the Caco-2 cells into cells simulating the small intestinal epithelium (Pinto et al. (1983) was achieved by culturing the cells in monolayers in 12-well tissue culture plates (Corning Inc., USA). For this, Caco-2 cells were seeded at a density of 1.6 × 10^5^ cells/mL, and growth medium was changed every 2 or 3 days. These cells are known to be fully differentiated after being cultured for 14 days.

### Simulated gastrointestinal passage (see Figure 2)

Before the start of the experiment, the pH of the SGF was re-checked and reset at 2.5 ± 0.1 using hydrochloric acid (1.0 mol/L). From an overnight bacterial culture (ON), 1 ml was mixed with 9 ml SGF, and incubated for 30 min at 37°C in a humidified atmosphere of 95% air and 5% CO_2_. Subsequently, 4 ml SGF/strain-mixture was mixed with 40 ml SIF-complete, and incubated for 2 h at 37°C in micro-aerophilic conditions (6% O_2_) (Anoxomat, Mart Microbiology, The Netherlands) on an orbital shaker at ~50 rpm. Further interaction of the SIF/strain-mixture with intestinal cells is described below (attachment and invasion assay). From each step in the gastrointestinal passage, an aliquot was used for enumerating the number of surviving bacteria.

**Figure 2:**
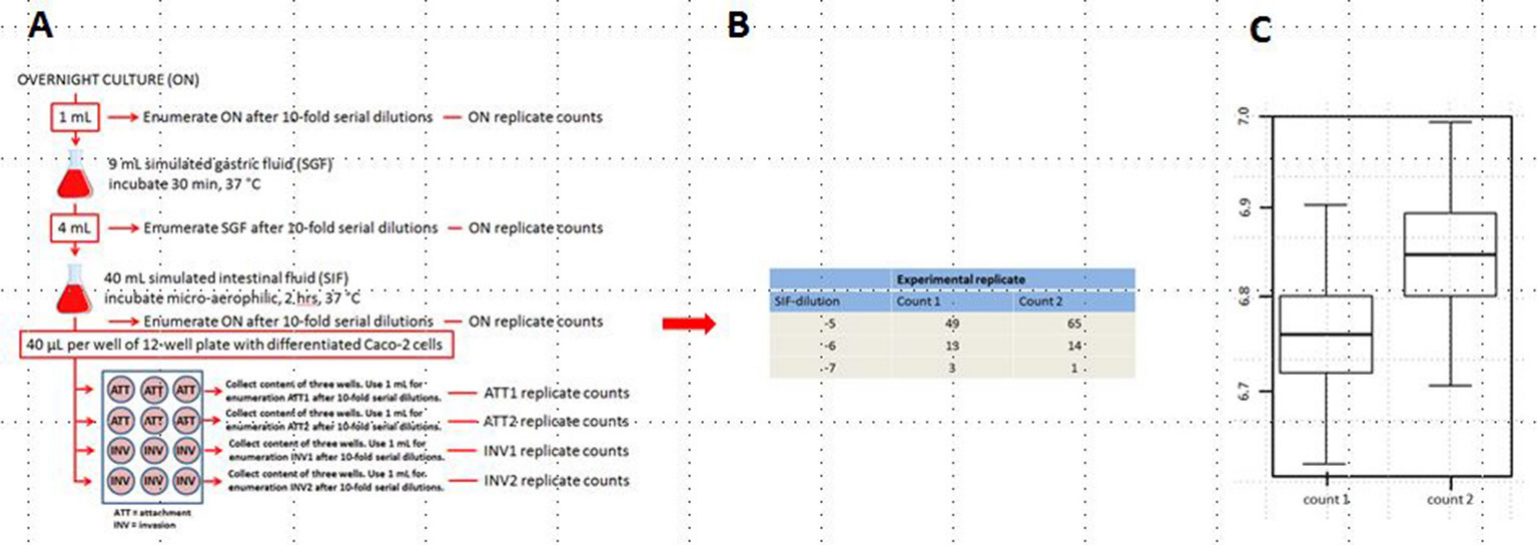
Schematic set-up of the simulated GIT-passage system **(A)**, replicate counts occur in all stages ON, SGF, SIF, ATT1, ATT2, INV1, and INV2 **(B)**, an example of the plate counts is given for SIF. Subsequent example of uncertainty in the natural log concentration estimates for count1 and count2 **(C)** in the SIF samples. Similar counts (as **B**) and concentration estimates expressed in boxplots (as **C**) are produced for all individual stages: ON, SGF, SIF, ATT1, ATT2, INV1, and INV2.

### Attachment and invasion assay (see Figure 2)

The method used for studying the rate of epithelial attachment (ATT) and invasion (INV) was based on previous work by Berk (2008). Prior to attachment and invasion assays, Caco-2 cells were washed three times with sterile phosphate-buffered saline (PBS, bioTRADING Benelux B.V., The Netherlands) to remove traces of antibiotic. After the final washing, 1 mL prewarmed DMEM without FBS and gentamicin (ECM, experimental culture medium) was added to each well. Afterwards, each well of the plates was inoculated with 40 μL SGF/SIF/strain-mixture per well (per strain all wells of a 12-well plate were inoculated). The plates were incubated at 37°C in a humidified atmosphere of 95% air and 5% CO_2_ during 1 h for attachment assay. After incubation, the medium was aspirated and the monolayers were rinsed three times with PBS in order to remove non-attached/loosely attached bacteria. Subsequently, the cells were used for two purposes, namely either determination of the number of attached and invaded bacteria, or determination of the number of invaded bacteria.

For enumerating the number of attached and invaded bacteria, Caco-2 cells in 6 of the 12 wells were lysed (in order to liberate the bacteria) with 1 mL 1% (v/v) Triton-X100 (Merck) in PBS, for 5 min at room temperature. Twice, the Triton lysate from three wells was combined and the two lysates were named ATT1 and ATT2.

For quantifying the number of invading bacteria, the cells in the other six 6 wells of the 12-well plate were treated with ECM supplemented with 0.3% gentamicin (50 mg/mL, Gibco). (Data confirming the proper activity of this concentration of gentamicin during 3 h are not shown.) Gentamicin does not affect differentiated Caco-2 cells or invaded bacteria, but will inactivate attached bacteria (Berk, 2008). The plates were incubated for 3 h at 37°C in a humidified atmosphere of 95% air and 5% CO_2_. After incubation, the cells were washed three times with PBS to remove excess antibiotic and lysed with 1% (v/v) Triton-X100 to liberate invaded bacteria. Twice, the Triton lysate of three wells was used for determining the number of *Salmonella* that invaded the Caco-2 cells. The two lysates were named INV1 and INV2.

### Enumeration of bacteria in the stages of the GIT-system (see Figure 2)

From the overnight (ON), SGF and SIF stages of the GIT system, a single sample was investigated to determine the bacterial load. At the attachment and invasion stages two samples were investigated for their bacterial load (ATT1 and ATT2, respectively, and INV1 and INV2, respectively). After appropriate 10-fold serial dilutions, each sample was plated in duplicate on TSA.

The output of the GIT-simulation is from the first three stages (ON, SGF, and SIF) duplicate counts for each appropriate dilution (as shown in Figure 2B). The output for the attachment and invasion stages is duplicate counts for each appropriate dilution for ATT1, ATT2, INV1, and INV2, respectively. The uncertainty in the natural log concentration estimates for count1 and count2 is expressed in boxplots (Figure 2C).

## Statistical analysis

To estimate the changes in log concentration of inoculated salmonellae in all successive stages of the GIT system, a hierarchical Bayesian framework was set-up using Markov chain Monte Carlo (MCMC) from bacterial counts as described in Section Enumeration of Bacteria in the Stages of the GIT-System. Table 1 shows which strains were included in each experiment executed at 3 separate days.

**Table 1:** Design of the experiment days and investigated samples.

**Experiment day 1**		**Experiment day 2**		**Experiment day 3**	
**Source**	**Strain**	**Source**	**Strain**	**Source**	**Strain**
poultry	SH^#^ 980	poultry	SH 980	poultry	SH 980
child	SH 1007	child	SH 1007-1^*^	child	SH 1007
		child	SH 1007-2^*^		
child	SH 1011				
child	SH 1028				
		pasta	SH 1043-1^*^	pasta	SH 1043
		pasta	SH 1043-2^*^		
	STM^#^ 3283		STM 3283		STM 3283

# *SH, Salmonella Heidelberg; STM, Salmonella Typhimurium*.

* *−1 and −2 refers to a biological biological replicate of the same strain within 1 day*.

**Figure 3:**
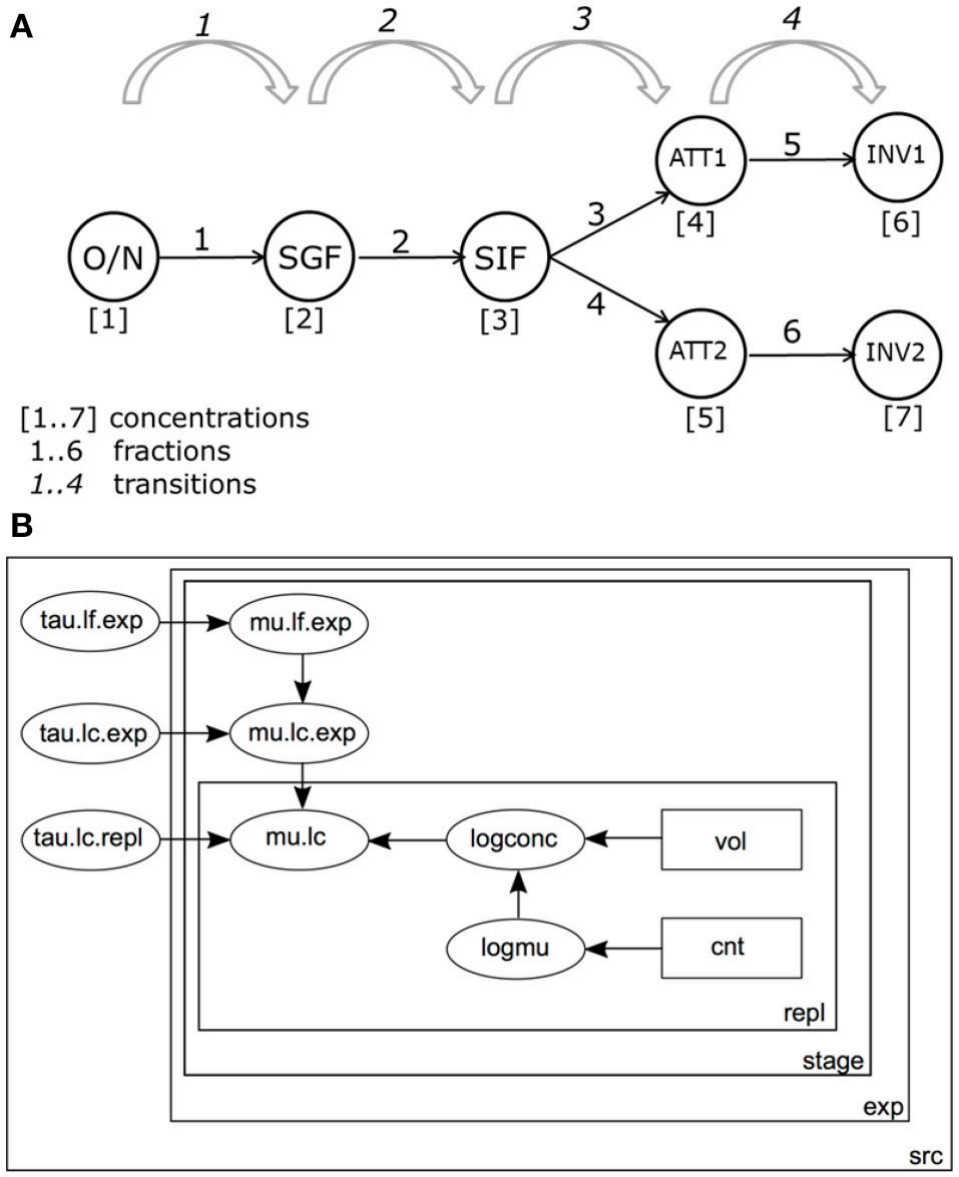
**(A)** Schematic representation of the *in vitro* GIT system. Bacterial counts are performed at the following stages during passage: ON, Overnight culture; SGF, Simulated Gastric Fluid; SIF, Simulated Intestinal Fluid; ATT1, Attachment to a first set of Caco-2 cells; ATT2, Attachment to a second set of Caco-2 cells; INV1, Invasion into a first set of Caco-2 cells; INV2, Invasion into a second set of Caco-2 cells. **(B)** Graph of the model for estimating log changes in the GIT system. Bacterial counts (cnt) in volumes (vol) are used to estimate separate log concentrations (logconc) for each replicate (repl) and accompanying expected log concentrations (logmu). By convention, precision τ (tau) is used instead of standard deviation ($\sigma =1/\sqrt{\tau }$). Replicate counts counts were used to calculate Log concentrations (mean mu.lc, precision tau.lc.repl). Concentration estimates from different experiments were combined, with mean mu.ls.exp, and precision tau.lc.exp. Concentrations by stage (stage) in the GIT system (Figure 2A), were calculated starting from ON and accounting for successive (log) changes *D (*mean mu.lf.exp, precision tau.lf.exp). All parameters were estimated separately for each strain-substrate combination (src).

Figure 3A shows a schematic representation of the GIT system and the different variables as used in the Bayesian framework. Both bacterial concentrations and log changes throughout the different stages of the GIT system were monitored in the model.

Bacteria pass through 4 stages in the GIT model: from overnight culture (ON) they are inoculated into SGF; after incubation, a sample is transferred into SIF. Subsequently, bacteria are inoculated onto a (confluent) culture of intestinal epithelial cells and numbers attached (ATT) are determined. Finally, the numbers of bacteria invading the intestinal cells are determined (INV). The final two stages are done in duplicate, resulting in 3 + 2^*^2 = 7 compartments where bacteria are enumerated (Figure 3A).

In each compartment, two samples are taken and individual counts were assumed Poisson.

$$\begin{array}{l} k\sim Pois\left(cV\right) \end{array}$$

with a concentration *c* in equivalent sample volume *V*, where the concentration in overnight culture is lognormally distributed

$$\begin{array}{l} log\left(\text{c}\right)\sim N\left(\mu_{lc},\sigma_{lc}\right) \end{array}$$

with mean μ_*lc*_ and standard deviation σ_*lc*_. The concentrations in successive stages may then be characterized by the log change (*D*) in concentration

$$\begin{array}{l} log\left(c_{n}\right)=log\left(c_{n-1}\right)+D_{n} \end{array}$$

where the log change is again normally distributed

$$\begin{array}{l} D_{n}\sim N\left(\mu_{n},\sigma_{n}\right) \end{array}$$

Note that the log change may be positive: bacteria may grow, for instance when incubated into SIF.

For each strain/substrate combination there is a starting (log) concentration and 4 (log) change factors (SGF/ON; SIF/SGF; ATT/SIF; INV/ATT). The probability of infection, P_inf_, can then be calculated as the product of the four transitions (Figure 2A) or, as log changes

$$\begin{array}{l} \log\left(P_{\text{inf}}\right)=\sum_{n=1}^{4}D_{n}. \end{array}$$

Figure 3B shows the structure of the statistical model for the practical GIT system.

The model distinguishes variation between replicate counts of the same sample within each experiment (Figure 3B), variation between biological experiments and variation between strain/substrate combinations (both in Table 1).

Experimental variability is inherent to the test system. For example, composition of the gastric and intestinal fluids, properties of the (Caco-2) cultured intestinal cells, and any human handling may differ between replicates within the same experiment and between biological experiments.

Biological variability is inherent to the test strain and its environment. For example, the number of bacteria in the overnight culture differs per experiment resulting in different dynamics when exposed to the GIT system. In addition, there is environmental variability affecting strain dynamics.

The model in Figure 3B was implemented in JAGS v4.2.0 (Plummer, 2003) run within R (v3.2) using the runjags library (v2.0.4-2), 30,000 posterior iterations, burnin 10000, thinning, to leave 3,000 posterior samples for further analysis.

## Results

### Quantifying variability in replicate counts for each test strain within each experiment

Variability of the test system within any experiment is expressed through different plate counts and corresponding concentration estimates. The estimated lognormal distribution of concentration represents the uncertainty about the true concentration in each sample based on these replicate counts. Variation between replicate counts should be small, and not exceed the (Poisson) uncertainty in concentration (expressed in boxplots in Figure 2C). Therefore, replicates were tested for consistency in the bacterial plate counts for each strain at each of the seven stages on the 3 separate days.

The statistical test for difference in counts was performed by comparing replicate measurements, using posterior MC samples by strain and stage in the GIT system. For testing, MC samples from replicate counts were subtracted and the fraction of the difference samples >0 was interpreted as a *p*-value for a meaningful difference (Gelman et al., 2013). A fraction smaller than 0.025 would indicate that the majority of the estimated concentrations from the second count was larger than that from the first count (and *vice versa* for a fraction >0.975).

**Figure 4:**
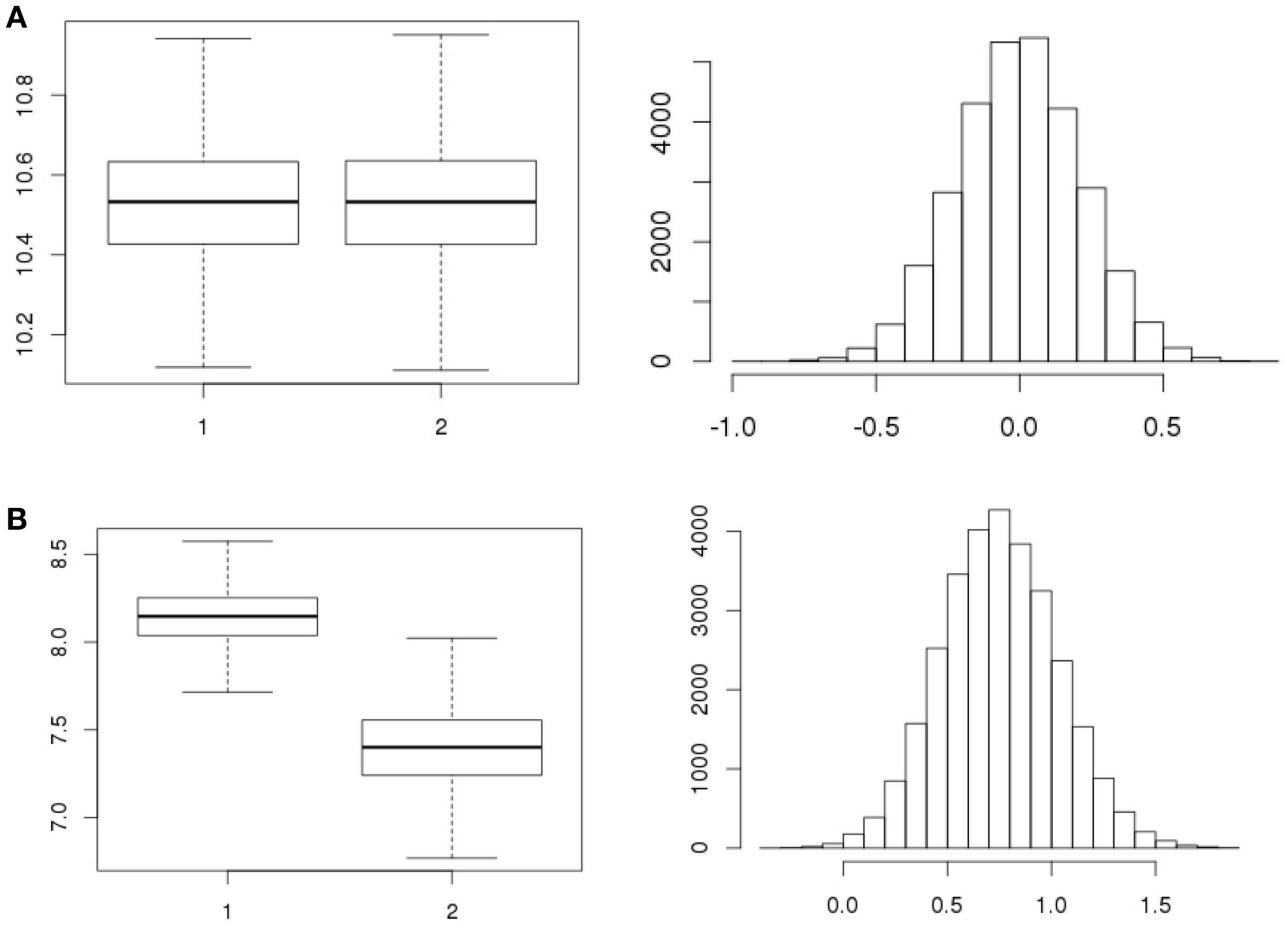
Example boxplots for the concentrations (natural logarithm) of replicates and corresponding histograms of the subtraction of the concentrations as used to quantify the difference in replicates for strain 1011 (**A**, exp.1 ATT1, *p* = 0.506 and **B**, exp.1 INV2, *p* = 0.998, Table 1).

A total number of, respectively, 5, 4, and 4 strains were tested in replicates over the three separate days (Table 1) for all 7 stages resulting in 91 statistical tests for difference in concentration. There were 12 tests revealing meaningful different replicate concentrations in the 91 replicate experiments, i.e., *p* < 0.025 or > 0.975. That is, for strain 980 in the ON and ATT stage; strain 1007 in the ON, SGF and 3 tests in the INV stage; strains 1011, 1028, 3283 in the INV stage; and strain 1043 for 2 tests in the INV stage. As an example, Figures 4A,B shows boxplots of consistent (a) and different (b) replicates in concentration estimates and corresponding histograms to visualize the subtraction of the MC sample replicates used to calculate the *p*-value in the statistical test for difference.

Biological variability in test results of similar strains when exposed to the GIT system on different days was modeled by the parameter tau.lc.repl (Figure 3B), accounting for the variation between replicate counts. One needs to account for this type of experimental variability if the ultimate goal is to assess the true biological variability between strains.

### Quantifying variability in counts of the same strain in replicate experiments on a given (same) day

Biological replicates were investigated during a second set of experiments for strains 1043 and 1007. No significant differences in change in log concentration over the different stages SGF/ON, SIF/SGF, ATT/SIF, and INV/ATT were identified for the biological replicates of strains 1043 and 1007 within 1 day. Strain 1007 had a *p*-value of 0.057 for the change in log concentration in SIF compared to SGF, all other *p*-values were in the range from 0.382 to 0.788 (individual data not shown).

### Quantifying variability in counts of the same strain in replicate experiments conducted on different days

Biological replicates were investigated over two separate experiments for strain 1043 and over 3 separate experiments for strains 980, 1007, and strain 3283. Estimates by experiment were compared as described above (see Section Quantifying Variability in Replicate Counts for Each Test Strain within Each Experiment) for the replicate counts. In order to account for differences in ON between experiments, results on changes in log concentration throughout the GIT system (the 6 fractions in Figure 3A, calculated as explained in Section Statistical Analysis) were used for statistical analysis.

In total 60 different tests (i.e., 10 day to day comparisons for the 4 strains over 6 fractions) were done to compare between days difference in log concentration change of strains 980, 1007, 1043, and 3283 for the 6 fractions. No differences in log concentration change of biological replicates between test days were found (data not shown).

### Quantifying uncertainty and variability in microbial infectivity risk estimates

Ultimately, one would like to use the *in vitro* system to make statements about difference in infectivity between different bacterial strains. As indicated in the introduction, the fraction of bacteria in the overnight culture that succeed in invasion of cultured intestinal cells can be considered a measure for infection probability (P_inf_).

MC estimates of P_inf_ show variation, resulting from experimental uncertainty and biological variation at each stage in the GIT system. Only when accounting for experimental uncertainty and biological variability *within* strains valid statements can be made about true biological differences between different strains with respect to survival through different stages of the GIT system and ultimate P_inf_. Moreover, *within within* strain experimental uncertainty and biological variability should also be taken into account when making predictions about infectivity based on results from a single biological experiment.

**Figure 5:**
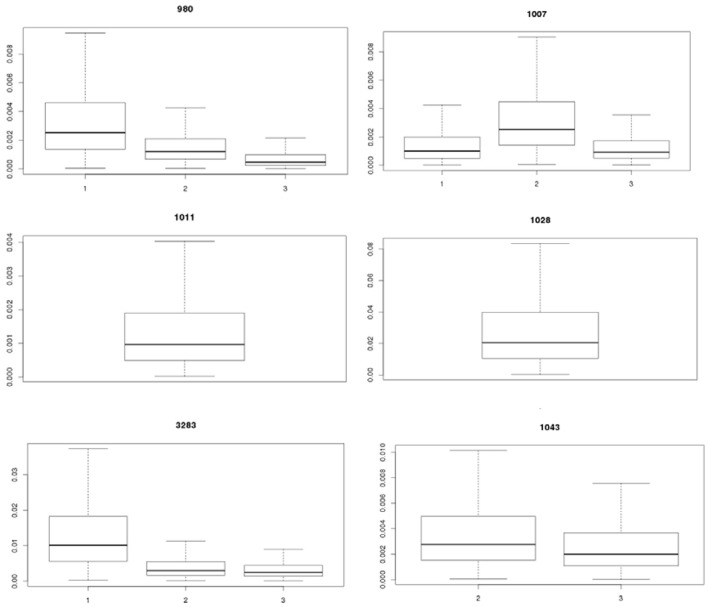
Visualization of the experimental uncertainty obtained from the replicate counts in each single biological experiment for strains 980, 1007, 1011, 1028, 3283, and 1043 on day 1, 2, and 3. The two biological experiments for strain 1007 (1007-1 and 1007-2) and for strain 1043 (1043-1 and 1043-2) within day 2 were merged to come to an overall estimate on experimental day 2 for both strains (biological experiments according to Table 1).

Table 2 shows the statistics for P_inf_ resulting from each separate biological experiment. Accompanying boxplots are presented in Figure 5.

**Table 2:** Expected P_inf_, median, standard deviation and 95% credible interval describing the experimental uncertainty obtained from the replicate counts in each single biological experiment.

					**Credible interval**	
**Strain**	**Experiment**	**Expected**	**Median**	**Standard deviation**	**Lower 0.025**	**Upper 0.975**
980	1	3.87E-03	2.78E-03	4.46E-03	6.08E-04	1.38E-02
980	2	1.80E-03	1.34E-03	1.77E-03	3.03E-04	6.18E-03
980	3	8.26E-04	6.01E-04	1.09E-03	1.31E-04	2.91E-03
1007	1	1.80E-03	1.26E-03	2.20E-03	2.32E-04	6.60E-03
1007	2	3.75E-03	2.74E-03	3.83E-03	6.16E-04	1.29E-02
1007	3	1.45E-03	1.09E-03	1.58E-03	2.50E-04	4.76E-03
1011	1	1.91E-03	1.22E-03	2.26E-03	1.91E-04	7.65E-03
1028	1	3.97E-02	2.52E-02	4.25E-02	4.21E-03	1.62E-01
3283	1	1.55E-02	1.13E-02	1.66E-02	2.43E-03	5.33E-02
3283	2	4.63E-03	3.48E-03	5.29E-03	7.94E-04	1.53E-02
3283	3	3.82E-03	2.86E-03	4.12E-03	6.87E-04	1.24E-02
1043	2	4.42E-03	3.19E-03	4.60E-03	6.84E-04	1.54E-02
1043	3	3.30E-03	2.38E-03	3.44E-03	5.10E-04	1.16E-02

Table 2 (and Figure 5) shows that the P_inf_ can roughly differ up to a factor 5 between estimated expected values for one particular test strain due to within strain variability over test days (compare strain 980 in experiment 1 and 3).

**Figure 6:**
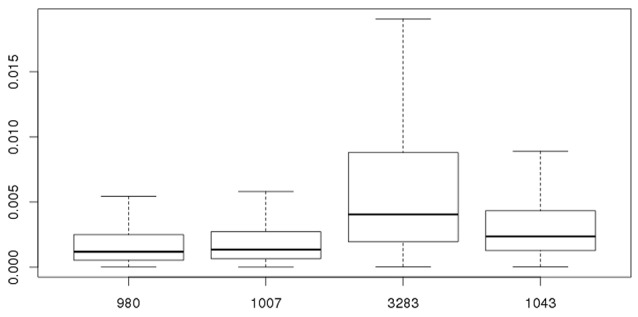
Overall probability of infection (P_inf_) for strains 980, 1007, 3283, and 1043. The 95% credible intervals include both the experimental uncertainty and between day biological variability of the underlying experiments.

As stated before, the biological variability within strains needs to be taken into account combined with experimental uncertainty when comparing different strains for P_inf_. Table 3 shows the statistics for the P_inf_ as an average over replicate biological experiments per strain (this excludes strains 1011 and 1028). Figure 6 visualizes Table 3 in which the boxplots include both the uncertainty of the underlying experiments and the between days biological variability.

**Table 3:** Expected P_inf_, median, standard deviation and 95% credible interval describing the experimental uncertainty and biological variability obtained as overall estimate from the merged counts in each single biological experiment.

					**Credibility interval**	
**Strain**	**Experiment**	**Expected**	**Median**	**Standard deviation**	**Lower 0.025**	**Upper 0.975**
980	1,2,3	2.11E-03	1.18E-03	3.10E-03	1.03E-04	9.66E-03
1007	1,2,3	2.24E-03	1.35E-03	2.88E-03	1.62E-04	9.70E-03
3283	1,2,3	7.70E-02	4.05E-02	1.16E-02	5.45E-04	3.71E-02
1043	2,3	3.55E-03	2.36E-03	4.10E-03	3.92E-04	1.40E-02

The model as shown in Figure 3B has a hierarchical structure, with nested sources of variation (replicate counts, dilutions, experiments). It is instructive to simplify the results, by assuming that experimental uncertainty from replicate counts/dilutions and biological variation from replicate experiments can be separated. If the total variance is the sum of two components, one representing experimental uncertainty and another representing biological variation, and these two components may be assumed independent stochastic variables, basic rules for variance calculations apply. Table 4 shows the average standard deviations for experimental uncertainty about P_inf_ for strains 980, 1007, 3283, and 1043 as calculated from the individual standard deviations in Table 2 following

$$\begin{array}{l} \frac{1}{3}\sqrt{\left(\sigma_{exp1}^{2}+\sigma_{exp2}^{2}+\sigma_{exp3}^{2}\right)}. \end{array}$$

Table 4 also shows the calculated standard deviation for biological variability about P_inf_ separately following

$$\begin{array}{l} \sqrt{\sigma_{unc\&biol.var.}^{2}-\sigma_{unc}^{2}} \end{array}$$

and the corresponding fractions as part of the total variability following

$$\begin{array}{l} \sigma_{unc}^{2}/\sigma_{unc\&biol.var}^{2} \end{array}$$

for the experimental uncertainty and

$$\begin{array}{l} \sigma_{biol.var.}^{2}/\sigma_{unc\&biol.var}^{2} \end{array}$$

for the biological variability. Using this information, one may conclude that if a single biological experiment is conducted and the contribution of experimental uncertainty (expressed in a standard deviation) to the expected P_inf_ is estimated, one needs to multiply the variance (describing uncertainty) with a factor of about 2.5 (e.g., 72/28). In order to account for biological variability (i.e., if the strain was to be tested again in another experiment on a different day), the result must be added to that variance (describing uncertainty).

**Table 4:** Expected P_inf_, calculated standard deviations for experimental uncertainty (unc.) about P_inf_, for biological variability (biol. var.) about P_inf_, an overall standard deviation about P_inf_ (combining unc. and biol. var.) and corresponding fractions of the overall standard deviation that can be attributed to unc. and biol. var. for strains 980, 1007, 3283, and 1043.

**Strain**	**Exp**.	**P_inf_ Expected**	**Standard deviation (unc.)**	**Standard deviation (biol.var.)**	**Standard deviation (unc. & biol.var.)**	**Unc. & biol. var. as fraction (%) of total variability unc. biol. var.**	
980	1,2,3	2.11E-03	1.64E-03	2.63E-03	3.10E-03	28	72
1007	1,2,3	2.24E-03	1.56E-03	2.42E-03	2.88E-03	29	71
3283	1,2,3	7.70E-02	5.97E-03	9.95E-03	1.16E-02	26	74
1043	2,3	3.55E-03	2.87E-03	2.93E-03	4.10E-03	49	51

**Figure 7:**
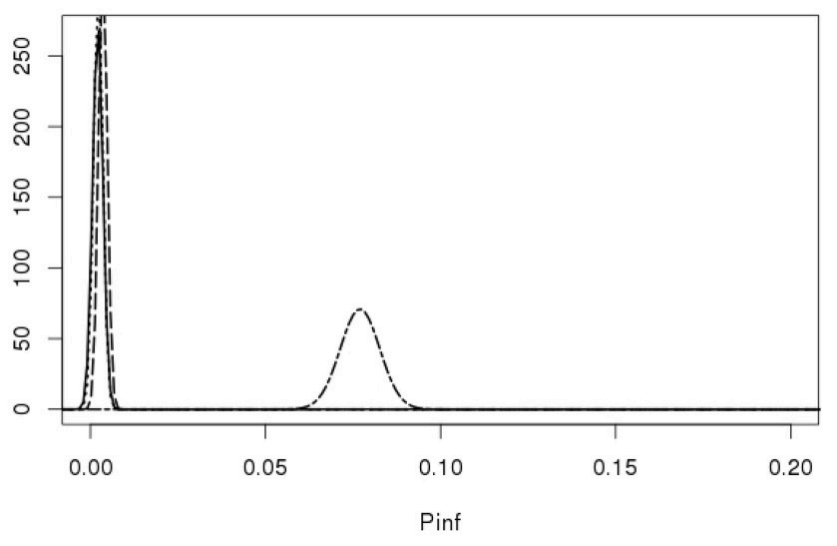
Lognormal distributions describing the biological variability of P_inf_ for the *S*. Heidelberg Heidelberg strains 980 (solid line), 1007 (dotted line), 1043 (dashed line) on the left and the *S*. Typhimurium strain 3283 (dot-dashed line) on the right. Parameters for the Lognormal distribution were calculated using the expected values and a standard deviations from Table 4 as input parameters, i.e., P_inf_ ~Lognormal(μ,σ) for strain 980: (−6.36, 0.62), 1007: (−6.25, 0.55), 1043: (−5.70, 0.34), and 3283 (−2.57, 0.07).

Figure 7 shows that the GIT system can quantify *true* biological variability between the different *Salmonella* Heidelberg strains and the *Salmonella* Typhimurium strain.

## Conclusions

The gastrointestinal tract system (GIT) developed in our laboratory allows quantification of pathogen survival and attachment/invasion into human intestinal mucosal cells. We have demonstrated that the estimated probabilities of survival can be quantified for all stages of the GIT system, and we have characterized the uncertainty associated with the estimated probabilities of survival. The GIT system has been demonstrated to identify biological differences between strains/serovars, when accounting for experimental uncertainty and biological variability within pathogen strains. Strain 3283 shows reproducible results and will be used as reference strain in future studies.

Using the hierarchical Bayesian model, the performance of future test strains may be predicted, that may be tested only once (in a single experiment) taking into account:

1. Experimental uncertainty of the plate counts for that single experiment.
2. Biological variability between test days based on the analysis in this paper.

The results of the GIT model can be used to determine the relative variation in infectivity *in vitro* as a proxy for *in vivo* variation in human DR. Including credible intervals (C.I.s) for the changes in log concentration gives insight in biological differences between test strains. This creates opportunities for aggregated risk ranking (grouping strains with overlapping C.I.'s) instead of arbitrarily ranking individual strains based on a point estimate (like the mean probability of infection). Including experimental uncertainty and within strain biological variability in the behavior of foodborne pathogens will also help understanding in current developments in explaining phenotypic behavior based on omics data (e.g., whole genome sequencing).

## Discussion

Here we describe a standardized model system for simulating the gastrointestinal (GIT) passage of foodborne pathogens, including the interaction with epithelial cells, represented by differentiated Caco-2 cells. With this model, we are able to compare the dynamics of different genera, serotypes or strains of foodborne bacteria in a standardized manner. The outcome is described as probability of infection (P_inf_). Dependent on the type of action of the foodborne bacteria, P_inf_ is determined by the fraction foodborne pathogens invading Caco-2 cells or the fraction of bacteria attaching to and invading Caco-2 cells. The probability of infection (P_inf_) is a measure for comparing infectivity of strains/serovars/genera of bacteria, thus, describing relative virulence.

By investigating multiple strains of *Salmonella* Heidelberg and *S*. Typhimurium at any given day and/or one single strain multiple times per day or over several days, both experimental uncertainty and biological variation in P_inf_ were determined. With this system, we can further study the potential cause of the relative pathogenic effects in DR, like the influence of genetic determinants on infectivity.

The main goal of these investigations was to estimate the measurement error in the log changes in pathogen concentration (possibly translated into transition probabilities) in the *in vitro* system, in order to interpret outcomes. Are differences in the estimated fractions (foodborne pathogens invading Caco-2 cells or attaching to and invading Caco-2 cells) likely to have been caused by biological differences between pathogen strains, or could they have been caused by measurement error and within strain biological variability: in short, determination of the robustness of the system.

Most differences in plate counts between experimental replicates were found at the stage where bacterial cells are tested for invasion into Caco-2 cells. Variation between replicate observations at the invasion stage could be explained by the added biological variation resulting from interaction of Caco-2 cells with the bacterial cells in the suspension used for enumeration. Turbidity of the suspension caused by inhomogeneous distribution of the bacterial cells may have resulted in inconsistent plate counts. This cause of experimental variability may be reduced through an improvement in the test system with respect to homogenization of the test suspension in the invasion stage.

Experimental uncertainty in the plate counts of the attachment and invasion part of the system can be reduced by improvement of the Caco-2 cell system which will result in less variable replicates, even though uncertainty will always exist, if only due to Poisson counts. These improvements could consist of a longer homogenization step before diluting the Triton-extracts, or leaving the Triton-extracts for half an hour at refrigeration temperature before starting homogenization, dilution and plating.

Biological variability between test days cannot be reduced by increasing the numbers of replicates. However, improved control of experimental procedures by repeatedly using the same equipment may decrease contribution of experimental error to the observed variation. Any biological variation caused by intrinsic changes in the pathogenic bacteria and/or the host cell populations cannot be reduced, and may be considered to contribute to the *in vivo* DR of the pathogenic bacteria in humans.

Another option would be to use all Markov chain iterations for ranking. Each posterior P_inf_ estimate would result in a ranking, e.g., 3,000 posterior estimates would result in 3,000 rankings per strain. Comparing overlap in these rankings between strains gives insight in statistically valid rankings.

The characterization of (experimental) uncertainty may be improved over time, by using this methodology in an iterative way. The Bayesian framework provides a straightforward means for improvement: new data may be included as they become available, updating current estimates to generate more accurate outcomes based on accumulating information.

Here, all shown calculations involved P_inf_, the overall probability of invasion, given overnight culture of a pathogen cell. Since data are known from all stages in the GIT-system, all these stages can be investigated separately, by the same approach. Thus, differential characterization of overnight culture, survival/growth in gastric fluid or intestinal fluid, and/or attachment to intestinal culture cells, is possible. This may be of interest for describing the association of genetic pathogen factors with specific phenotypic properties in the GIT system.

The GIT-system described in this paper has a number of advantages. In a single experimental day, one can investigate six to eight different strains, including one standard strain as a control. Such throughput cannot be achieved in human volunteers, test-animals or other simulation systems for the investigation of behavior of pathogenic microorganisms, like the TIM-model (Minekus, 2015) or the SHIME-system (Van de Wiele et al., 2015). The GIT system is much cheaper to use, and results are faster available. Moreover, this simulation model is also suitable for the investigation of any pathogenic bacteria that will attach to or invade human Caco-2 cells, which is certainly not possible with human volunteers or, within limits, with test animals.

The model system in its present form as described here, is meant for comparison of virulence of strains/serovars, resulting in relative virulence descriptions. Comparison of the results obtained with this new system with existing, and preferably, validated model(s) would improve the value of this new method. For this a few options exist. Firstly, this new model could be compared with existing animal models. However, for ethical reasons, namely reduction of the use of experimental animals, this option is not favored by the authors. In fact, the reduction of the use of experimental animals was one of the main reasons to develop this new system. Another possibility is comparison of this new system with existing experimental models, such as the TIM-model (Minekus, 2015). For this option one has to bear in mind that in our system, several strains can be compared at the same time, while the TIM-model is designed to determine the response of one strain at a time. In addition, there is another issue at stake. We have carried out many DR-studies, some of which based on natural experiments (outbreaks, Teunis et al., International Journal of Food Microbiology 2010, 144), and we attribute part of the variation of virulence in humans to the variation observed in this *in vitro* system, which sets limits on the range in virulence.

Currently the system is in use for comparing the behavior of different strains of different *Salmonella* serovars and for strains of different serotypes of Shiga Toxin producing *Escherichia coli*. In future, the system could be adapted to investigate translocation of pathogens (such as *Listeria monocytogenes*) through the epithelial layer, using Transwell® systems.

The model, as described in this paper, is not an exact reflection of differences in virulence between serovars *in vivo*. Various factors that could contribute to the survival or die-off of pathogenic bacteria are not (yet) incorporated in this model. Among these missing factors is mechanical stress due to movement of chyme through the intestinal tract, the presence of intestinal microbiota, and the influence of food components. The latter two factors and the influence of these on survival/die-off/attachment and invasion properties are currently under study. For the model as presented here, we do not foresee the incorporation of mechanical stress through tubing and valves. Other systems, like the TIM model (Minekus, 2015) are better equipped for that. Moreover, incorporation of mechanical stress would alter the possibility of determining the relative virulence of several strains in one experiment in a short period. The incorporation of intestinal microbiota and the influence of this on survival/die-off/attachment and invasion properties are currently under study.

Thus, this model system can be used to investigate all three contributors of foodborne disease, the pathogen, the food matrix and the host.

## Author contributions

LW was responsible for initiating the investigations, large part of the practical work, and initiating the statistical work. ED and AK were responsible for a large part of the pratical work. PT was responsible for assistance with the statistical calculations. AP was project-leader and responsible for the statistical calculations and supervision of finance.

### Conflict of interest statement

The authors declare that the research was conducted in the absence of any commercial or financial relationships that could be construed as a potential conflict of interest.

## References

[B1] Berk P. A. (2008). In vitro and In vivo Virulence of Salmonella typhimurium DT104: a Parallelogram Approach. Ph.D. thesis, Wageningen University, Wageningen.

[B2] Gelman A. Carlin J. B. Stern H. S. Dunson D. B. Vehtari A. Rubin D. B. (2013). Bayesian Data Analysis, 3rd Edn. Boca Raton, FL CRC Press.

[B3] Haas C. N. Thayyar-Madabusi A. Rose J. B. Gerba C. P. (2000). Development of a dose-response relationship for *Escherichia coli* O157:H7. Int. J. Food Microbiol. 56, 153–159. 10.1016/S0168-1605(99)00197-X 10857541

[B4] Havelaar A. H. Garssen J. Takumi K. Koedam M. A. Dufrenne J. B. van Leusden F. M.. (2001). A rat model for dose-response relationships of *Salmonella enteritidis* infection. J. Appl. Microbiol. 91, 442–452. 10.1046/j.1365-2672.2001.01399.x 11556909

[B5] Kothary M. H. Babu U. S. (2001) Infective dose of foodborne pathogens in volunteers: a review. J. Food Saf. 21, 49–68. 10.1111/j.1745-4565.2001.tb00307.x.

[B6] Minekus M. (2015). The TNO gastrointestinal model (TIM), in The Impact of Food Bioactives on Health: In vitro and Ex vivo Models, eds Verhoeckx K. Cotter P. López-Expósito I. Kleiveland C. Lea T. Mackie A. Requena T. Swiatecka D. Wichers H. (Cham: Springer International Publishing), 37–46. 29787039

[B7] Oliveira M. Wijnands L. Abadias M. Aarts H. Franz E. (2011). Pathogenic potential of *Salmonella typhimurium* DT104 following sequential passage through soil, packaged fresh-cut lettuce and a model gastrointestinal tract. Int. J. Food Microbiol. 148, 149–155. 10.1016/j.ijfoodmicro.2011.05.013 21665311

[B8] Oomen A. G. van Twillert K. Hofhuis M. F. A. Rompelberg C. J. M. Versantvoort C. H. M. (2003). Development and Suitability of In vitro Digestion Models in Assessing Bioaccessibility of Lead from Toy Matrices. National Institute for Public Health and the Environment (RIVM), RIVM-report nr 320102001, Bilthoven.

[B9] Pinto M. Robine L. Appay M.-D. Kedinger M. Triadou N. Dussaulx E. (1983). Enterocyte-like differentiation and polarization of the human colon carcinoma cell line Caco-2 in culture. Biol. Cell. 47, 323–330.

[B10] Plummer M. (2003). JAGS: a program for analysis of Bayesian graphical models using Gibbs sampling, in Proceedings of the 3rd International Workshop on Distributed Statistical Computing (DSC) (Vienna), 1–10.

[B11] Rotard W. Christmann W. Knoth W. Mailahn W. (1995). Determination of absorption availability of PCDD/PCDF from “Kieselrot” (red slag) in the digestive tract. Umweltwissenschaften und Schadstoff-Forschung 7, 3–9. 10.1007/BF02938733

[B12] Stecher B. Macpherson A. J. Hapfelmeier S. Kremer M. Stallmach T. Hardt W.-D. (2005). Comparison of *Salmonella enterica* serovar *Typhimurium colitis* in germfree mice and mice pretreated woth streptomycin. Infect. Immun. 73, 3228–3241. 10.1128/IAI.73.6.3228-3241.2005 15908347 PMC1111827

[B13] Strachan N. J. C. Doyle M. P. Kasuga F. Rotariu O. Ogden I. D. (2005). Dose response modelling of *Escherichia coli* O157 incorporating data from foodborne and environmental outbreaks. Int. J. Food Microbiol. 103, 35–47. 10.1016/j.ijfoodmicro.2004.11.023 16084264

[B14] Teunis P. F. M. Chappell C. L. Okhuysen P. C. (2002). Cryptosporidium dose-response studies: variation between hosts. Risk Anal. 22, 475–485. 10.1111/0272-4332.00046 12088227

[B15] Teunis P. F. M. Kasuga F. Fazil A. Ogden I. D. Rotariu O. Strachan N. J. C. (2010). Dose response modeling of Salmonella using outbreak data. Int. J. Food Microbiol. 144, 243–249. 10.1016/j.ijfoodmicro.2010.09.026 21036411

[B16] Teunis P. F. M. Ogden I. D. Strachan N. J. C. (2008). Hierarchical dose response of *E. coli* O157:H7 from human outbreaks incorporating heterogeneity in exposure. Epidemiol. Infect. 136, 761–770. 10.1017/s0950268807008771 17672927 PMC2870861

[B17] Teunis P. F. M. van der Heijden O. G. van der Giessen J. W. B. Havelaar A. H. (1996). The Dose Response Relation in Human Volunteers for Gastro-Intestinal Pathogens. Bilthoven: National Institute of Public Health and the Environment, RIVM report nr 284 550 002.

[B18] Teunis P. Takumi K. Shinagawa K. (2004). Dose response for infection by *Escherichia coli* O157:H7 from outbreak data. Risk Anal. 24, 401–407. 10.1111/j.0272-4332.2004.00441.x 15078310

[B19] Van de Wiele T. Van den Abbeele P. Ossieur W. Possemiers S. Marzorati M. (2015). The simulator of the human intestinal microbial ecosystem (SHIME®), in The Impact of Food Bioactives on Health: In vitro and Ex vivo Models, eds. Verhoeckx K. Cotter P. López-Expósito I. Kleiveland C. Lea T. Mackie A. Requena T. Swiatecka D. Wichers H. (Cham: Springer International Publishing), 305–317. 29787039

[B20] Van Rijckevorsel G. G. C. Mieras L. Bovée L. P. M. J. van Dijk C. Scholing M. Swaan C. M. (2015). A foodborne outbreak of Salmonella Heidelberg in nurseries (in Dutch). Infect. Bull. 26, 118–120. Available online at: http://www.rivm.nl/Documenten_en_publicaties/Algemeen_Actueel/Uitgaven/Infectieziekten_Bulletin/Jaargang_26_2015/Juni_2015/Inhoud_juni_2015/Een_voedselgerelateerde_uitbraak_van_Salmonella_Heidelberg_op_kinderdagverblijven

